# Variations in arterial blood pressure are associated with parallel changes in FlowTrac/Vigileo^®^-derived cardiac output measurements: a prospective comparison study

**DOI:** 10.1186/cc8161

**Published:** 2009-11-09

**Authors:** Savvas Eleftheriadis, Zisis Galatoudis, Vasilios Didilis, Ioannis Bougioukas, Julika Schön, Hermann Heinze, Klaus-Ulrich Berger, Matthias Heringlake

**Affiliations:** 1 Department of Anesthesiology, University General Hospital of Alexandropoulis, Dragana, Alexandropoulis, PC 68100, Greece; 2 Department of Cardiothoracic Surgery, University General Hospital of Alexandropoulis, Dragana, Alexandropoulis, PC 68100, Greece

## Abstract

**Introduction:**

The reliability of autocalibrated pressure waveform analysis by the FloTrac-Vigileo^® ^(FTV) system for the determination of cardiac output in comparison with intermittent pulmonary arterial thermodilution (IPATD) is controversial. The present prospective comparison study was designed to determine the effects of variations in arterial blood pressure on the reliability of the FTV system in patients undergoing coronary artery bypass grafting (CABG).

**Methods:**

Comparative measurements of cardiac output by FTV (derived from a femoral arterial line; software version 1.14) and IPATD were performed in 16 patients undergoing elective CABG in the period before institution of cardiopulmonary bypass. Measurements were performed after induction of anesthesia, after sternotomy, and during five time points during graft preparation. During graft preparation, arterial blood pressure was increased stepwise in intervals of 10 to 15 minutes by infusion of noradrenaline and lowered thereafter to baseline levels.

**Results:**

Mean arterial blood pressure was varied between 85 mmHg and 115 mmHg. IPATD cardiac output did not show significant changes during periods with increased arterial pressure either during sternotomy or after pharmacological manipulation. In contrast, FTV cardiac output paralleled changes in arterial blood pressure; i.e. increased significantly if blood pressure was raised and decreased upon return to baseline levels. Mean arterial blood pressure (MAP) and FTV cardiac output were closely correlated (r = 0.63 (95% confidence interval [CI]: 0.49 - 0.74), *P *< 0.0001) while no correlation between MAP and IPATD cardiac output was observed. Bland-Altman analyses for FTV versus IPATD cardiac output measurements revealed a bias of 0.4 l/min (8.5%) and limits of agreement from 2.1 to -1.3 l/min (42.2 to -25.3%).

**Conclusions:**

Acute variations in arterial blood pressure alter the reliability of the FlowTrac/Vigileo^® ^device with the second-generation software. This finding may help to explain the variable results of studies comparing the FTV system with other cardiac output monitoring techniques, questions the usefulness of this device for hemodynamic monitoring of patients undergoing rapid changes in arterial blood pressure, and should be kept in mind when using vasopressors during FTV-guided hemodynamic optimization.

## Introduction

Autocalibrated pressure waveform analysis by the FlowTrac/Vigileo^® ^(FTV) system allows determination of cardiac output (CO) from the arterial pressure curve. Controversy exists about the reliability of this technique in comparison with intermittent pulmonary arterial thermodilution (IPATD), especially during cardiac surgery [1,2]. A recent meta-analysis came to the conclusion that "cardiac output values provided by the FloTrac/Vigileo^® ^operating systems with software version 1.07 or later show acceptable agreement with ITD (intermittent thermodilution), both clinically and statistically" [3]. This conclusion contrasts sharply with our own results [4] and observations from Compton and colleagues [5] showing more than 40% percentage error in CO measurements by FTV in comparison with IPATD or transpulmonary thermodilution with the PiCCO^®^-system.

When using the FTV-system in patients undergoing non-cardiac surgery and critically ill patients in the intensive care unit (ICU) we have frequently observed increases in CO following a bolus of a vasopressor titrated to achieve a normal mean arterial blood pressure (MAP). This is a unique finding and is in contrast to what is to be expected from physiology, because the normal CO response during an increase in afterload in a patient with preserved cardiac function would be a short-lasting decrease in stroke volume followed by restoration of stroke volume and CO to the previous level, but not an increase in CO [6].

The present study was thus designed to determine the effects of variations in arterial pressure on the reliability of CO measurements by autocalibrated pulse wave analysis with the FTV system in comparison with IPATD. With respect to the fact that pulmonary artery catheters are routinely used in the cardiac anesthesia department but not in the noncardiac surgery population, the study was performed in cardiac surgery patients undergoing coronary artery bypass grafting (CABG) in the period before cardiopulmonary bypass.

## Materials and methods

Following approval by the local ethical committee (Scientific council of the General Hospital of Alexandropoulis) and written informed consent, 16 consecutive patients (all male) scheduled for standard on-pump CABG with moderate hypothermia were enrolled (mean ± standard deviation: age: 62 ± 10 years, weight: 83 ± 11 kg; height: 167 ± 8 cm, left ventricular ejection fraction: 64 ± 10%) for this prospective comparison study. All patients had a three-vessel coronary artery disease, a history of arterial hypertension, and hyperlipidemia. Three patients had diabetes and one had a history of stroke.

Following premedication with oral diazepam, general anesthesia was induced with fentanyl and etomidate and maintained with propofol and remifentanyl, as appropiate. Endotracheal intubation was facilitated with cisatracurium. All patients were equipped with a five lead electrocardiogram, a femoral arterial line, a triple lumen central venous catheter and a pulmonary artery catheter connected to a Vigilance I monitor (Edwards Lifesciences, Irvine, CA, USA).

After induction of anesthesia the pulmonary artery catheter was floated into the pulmonary artery until a typical pressure profile was obtained. Thereafter, a FloTrac/Vigileo^® ^system (Edwards Lifesciences, Irvine, CA, USA) was connected to the femoral arterial line, the transducer was adjusted to the level of left atrium and the system was started according to the instructions of the manufacturer (including entering the requested demographical data of the patient).

In the further course, comparative measurements of CO by IPATD and the FTV system were performed. MAP was recorded concomitantly. Bolus thermodilution CO measurements were performed in triplicate to quadruplicate with 4°C cold saline and averaged for respective time points. In general, three thermodilution measurements were performed. If the difference between these measurements was greater than 0.5 l/min, an additional measurement was performed and the three most contiguous results were averaged.

Autocalibrated CO measurements from the FTV system were recorded immediately after a bolus of saline was given for thermodilution (resulting in three to four measurements each that were again averaged). To assure adequate pressure recordings the arterial line was repeatedly flushed with 5 ml saline throughout the observation period and observed for tracing quality.

Comparative measurements were performed after induction, after sternotomy, and in the period of graft preparation (GP1 to GP5) before cardiopulmonary bypass. During graft harvesting, arterial blood pressure was titrated in periods of 10 to 15 minutes from a stable baseline around 80 mmHg (GP1 and GP2) to 100 mmHg (GP3) and further to higher than 110 mmHg (GP4) by a continuous infusion of noradrenaline (2.6 μg/min to 6.6 μg/min). Thereafter blood pressure was allowed to decrease back to levels around 80 mmHg (GP5).

### Statistical analyses

Data analyses were performed by MedCalc 10.4 (MedCalc Software bvba, Mariakerke, Belgium). Following Kolmogoronov-Smirnov test for normal distribution, data were analyzed parametrically. Between group differences were analyzed by analysis of variance. Intraindividual changes were analyzed by paired Student's t-test with Bonferoni-adjustment. Correlation analyses were performed by linear regression. Comparisons between methods were performed by Bland-Altman statistics. A *P *< 0.05 was considered statistically significant.

## Results

**Figure 1:**
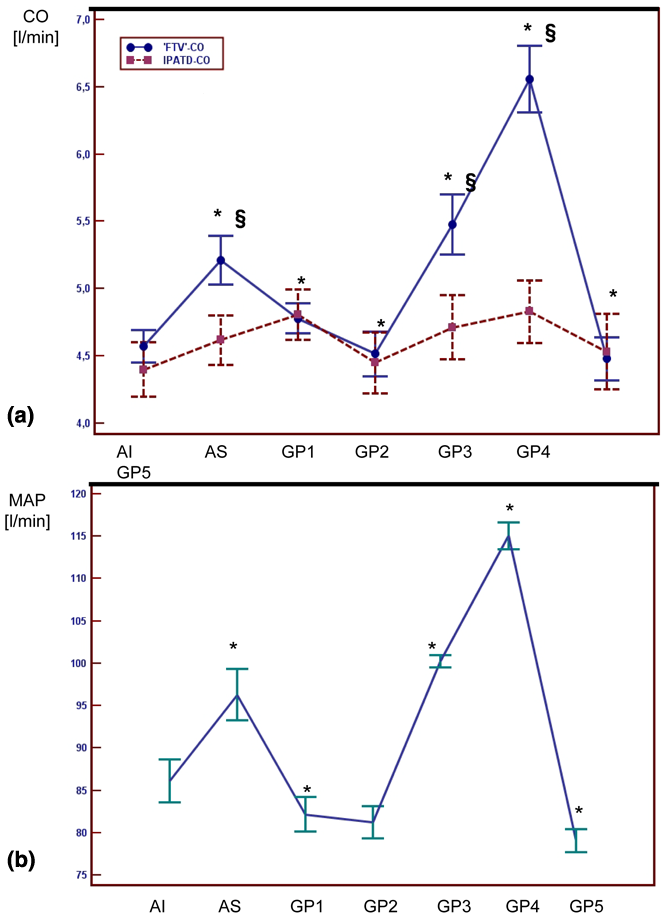
Cardiac output and mean arterial pressure during the study period. The time course of (a) cardiac output (CO) determined by intermittent pulmonary arterial thermodilution (filled circles = IPATD-CO) and autocalibrated pressure waveform analysis with the FlowTrac/Vigileo^® ^system (filled squares = FTV-CO) and (b) mean arterial pressure (MAP) in patients undergoing coronary artery bypass grafting surgery before cardiopulmonary bypass subjected to variations in arterial blood pressure either by the surgical stimulation or noradrenaline infusion. * significant difference (*P *< 0.05) in comparison with the previous time point (Student's t-test with Bonferoni-correction). § significant difference between IPATD-CO and FRV-CO (analysis of variance) AI = after induction; AS = after sternotomy; GP = graft preparation. CABG =; CI = confidence interval; CO = cardiac output; FTV = Flowtrac-Vigileo^®^; GP = graft preparation; IPATD = intermittent pulmonary arterial thermodilution; ICU = intensive care unit; MAP = mean arterial blood pressure.

The course of CO measurements and MAP is given in Figure 1, showing significant increases in MAP after sternotomy and during GP 3 and GP 4. No significant changes in IPATD cardac output were observed while FTV CO significantly increased during these blood pressure steps. Heart rate did not change significantly throughout the study period (data not shown).

**Figure 2:**
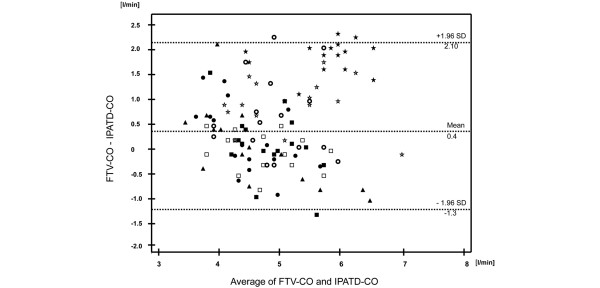
Bland-Altmann plot of absolute cardiac output data determined by intermittent pulmonary arterial thermodilution (IPATD-CO) and autocalibrated pressure waveform analysis with the Flowtrac/Vigileo^®^-system (FTV-CO) throughout the study. Closed circles = after induction; open circles = after sternotomy; closed squares = graft preparation 1; open squares = graft preparation 2; open stars = graft preparation 3; closed stars = graft preparation 4; closed triangles = graft preparation 5.

Correlation analysis revealed moderate correlations between FTV-CO and IPATD-CO (r = 0.51, 95% confidence interval (CI): 0.35 to 0.64, *P *< 0.0001) and between MAP and FTV-CO (r = 0.63, 95% CI: 0.49 to 0.74, *P *< 0.0001) but no correlation between MAP and IPATD-CO. Bland-Altman analyses for FTV-CO versus IPATD-CO revealed a bias 0.4 l/min and limits of agreement from 2.1 to -1.3 l/min for the pooled data (Figure 2). The respective percentage results were: bias 8.5%, limits of agreement 42.2% to - 25.3%.

Bland-Altman analyses at the individual data acquisition points are shown in Table 1, showing percentage errors higher than 30% at most measurement points and an increase in bias at the time points with raised MAP.

**Table 1:** Results of the Bland-Altman analyses at different time points

	AI	AS	GP1	GP2	GP3	GP4	GP5
**Raw data**							

Upper LoA (l/min)	1.46	2.2	1.27	1.28	1.74	2.63	1.6

Bias (l/min)	0.18	0.6	-0.02	0.07	0.74	1.73	-0.1

Lower LoA (l/min)	-1.11	-1.0	-1.32	-1.15	-0.21	0.83	-1.7

**Percentage data**							

Upper LoA (%)	36.1	44.6	28.8	31.5	34.0	48.0	38.3

Bias (%)	5	12.2	0.3	2.2	15.4	30.8	0.5

Lower LoA (%)	-26.1	-20.2	28.3	26.9	-3.2	13.5	-37.3

AI = after induction; AS = after sternotomy; GP 1 to 5 = graft preparation time points 1 to 5; LoA = limits of agreement (1.96 standard deviations).

## Discussion

Adequate monitoring of CO and stroke volume is a pivotal part of any hemodynamic optimization protocol and has traditionally been accomplished by using a pulmonary artery-catheter. And although it is now clearly established that the use of a pulmonary artery catheter is not associated with an increase in mortality, its use should be restricted to units with specialized knowledge and experience in using this technology [7]. Within the past years several alternative devices for the monitoring of CO have been developed and introduced into clinical practice.

One of the most recent developments is autocalibrated pressure waveform analysis by the FTV system [8]. The system differs from conventional pulse contour analysis systems (which are externally calibrated by bolus thermodilution) by using individual demographics, the skewness, and the kurtosis of the pulse to estimate arterial compliance and to adapt for changes in vascular tone. Following initial disappointing results [9] the software has undergone several refinements and the manufacturer now claims that it adapts every minute for changes in arterial compliance.

It is well known that changes in arterial resistance either by a vasodilating or a vasoconstricting agent may change pulse wave velocity and thereby influence peripheral as well as central aortic pulse contour [10]. In line with this, it has repeatedly been shown that conventional calibrated pulse contour CO monitors such as the PiCCO^® ^need repeated recalibration if such changes occur [11,12]. The present study was designed to determine if the FTV-system is robust against changes in vascular tone, that is an increase in vascular resistance induced by infusion of a vasopressor.

Our results clearly show that the autocalibration algorithm of the FTV system was not capable to adapt to changes in MAP between 80 to 110 mmHg (that were maintained for 10 to 15 minutes) although the software generation used calculates arterial compliance every minute: results in a percentage error between both methods that is clinically not acceptable. This is highly suggestive that the algorithm fails to detect short-term changes in systemic vascular resistance and may help to explain why the FTV-system has repeatedly been shown to underestimate CO in the immediate period after cardiopulmonary bypass or in patients with liver cirrhosis (i.e. during a vasodilatatory state with decreased vascular resistance [9,13]) but is capable of reliably detecting fluid induced changes in stroke volume (i.e. changes in preload that are typically not accompanied by immediate changes in vascular tone) [14] or pacing induced changes in CO [15]. Unfortunately, we did not use any direct and objective measures to determine vascular resistance (i.e. determination of forearm blood by strain gauge) and thus this explanation remains speculative.

It is of note, that the percentage error at most measurement time points was higher than 30% and that the FTV system *per se *did not reliably measure CO in comparison with IPATD, even if arterial blood pressure was in the normal range. This further questions the clinical usefulness of this device, at least with the software version used in this study.

## Conclusions

The results of the present study show that changes in systemic arterial resistance alter the reliability of the FTV-system; even if using the modified second-generation software (version 1.14). This may help to explain the variable results of studies comparing the FTV-system with other CO monitoring techniques, questions the usefulness of this device for hemodynamic monitoring of patients undergoing rapid changes in arterial blood pressure, and should be kept in mind when using vasopressors during FTV-guided hemodynamic optimization. Further studies are needed to reveal if the most recent modification of the FTV-system software (the third generation) improves the reliability of this technology.

## Key messages

• Variations in arterial blood pressure lead to parallel changes in CO measurements by the second generation of the FTV system.

• This questions the usefulness of this device for hemodynamic monitoring of patients undergoing rapid changes in arterial blood pressure and should be kept in mind when using vasopressors during Flowtrac/Vigileo^® ^- guided hemodynamic optimization.

## Abbreviations

CABG: coronary artery bypass grafting; CI: confidence interval; CO: cardiac output; FTV: FlowTrac-Vigileo^®^; GP: graft preparation; ICU: intensive care unit; IPATD: intermittent pulmonary arterial thermodilution; MAP: mean arterial blood pressure.

## Competing interests

The authors SE, ZG, VD, IB, JS, HH, and KUB declare that they have no competing interests. MH received scientific support and/or honoraria for lectures from Edwards Lifesciences, Irvine, CA, USA, the manufacturer of the FloTrac/Vigileo^® ^- system, Osypka Medical, Germany, and Covidien, Germany.

## Authors' contributions

SE, JS, and MH designed the study, performed the statistical analyses and drafted the manuscript. SE, ZG, VD, and IB coordinated the study, were responsible for patient recruitment and data acquisition. HH and KUB were involved in the interpretation of the data and manuscript drafting. All authors read and approved the final manuscript.
